# Rapid Far-Infrared Radiation and Physiotherapeutic Effects of Carbon Nanotube Flexible Thin-Film Heaters

**DOI:** 10.3390/nano16090539

**Published:** 2026-04-29

**Authors:** Shi-Yao Wang, Yue-Xin Wang, Wen-Zheng Li, Meng-Yao Li, Jia-Yi Gao, Pu Liu, Jing Zhou, Xuguo Huai, Hong-Zhang Geng

**Affiliations:** 1 Tianjin Key Laboratory of Advanced Fibers and Energy Storage, School of Material Science and Engineering, Tiangong University, Tianjin 300387, China; wangsy-2005@outlook.com (S.-Y.W.); 2420020100@tiangong.edu.cn (Y.-X.W.); lwz0217@outlook.com (W.-Z.L.); jialbhd_0711@outlook.com (M.-Y.L.); 2410220112@tiangong.edu.cn (J.-Y.G.); 2410220212@tiangong.edu.cn (P.L.); zj-zhouj@outlook.com (J.Z.); 2 Center for Engineering Internship and Training, Tiangong University, Tianjin 300387, China; 3 Hebei Province Technology R&D Platform, Hebei Industrial Technology Research Institute of Membranes, Cangzhou Institute of Tiangong University, Cangzhou 061000, China

**Keywords:** carbon nanotubes, flexible thin-film heaters, far-infrared radiation, physiotherapeutic efficacy, rapid response

## Abstract

Carbon nanotube (CNT) materials exhibit ultrahigh electrical and thermal conductivity. Upon electrical excitation, CNT-based transparent conductive films (TCFs) can emit far-infrared radiation (FIR) and provide certain physiotherapeutic efficacy, making them ideal candidates for thermotherapy applications. This work systematically tests and analyzes the fundamental physical properties and physiotherapeutic performance of CNT flexible thin-film heaters (TFHs) for potential use in health physiotherapy. Two types of TFHs with different electrode connection modes were fabricated via the prepared TCFs. Experimental characterizations were conducted on their response time, electrothermal performance, and heat transfer characteristics. The results showed that the temperature rise per unit input power for TFH1 was 16.71 °C/W, while that of TFH2 was 4.29 °C/W at the same voltage of 10 V. In addition, the variation trends of maximum temperature with power density were highly consistent for the two films. This demonstrates that TFHs fabricated using the same TCFs exhibit excellent and high electrothermal conversion efficiency as well as outstanding comprehensive electrothermal performance. In addition, smaller L/W ratio leads to lower resistance of TFHs, resulting in a stronger thermal effect under identical applied voltage. After the temperature stabilized, the surface temperature of the TFHs decreased by approximately 5 °C when attached to the human arm, confirming that the heat generated by the TFHs under electrical excitation could be effectively absorbed by the human body. The TFHs emitted rapid FIR upon electrification, and the peak wavelength ranged from 8 to 12 µm, which fell within the range of 6–14 µm that was easily absorbable by the human body. The heat can be rapidly absorbed by the skin and distributed throughout the body via blood circulation, yielding favorable physiotherapeutic efficacy. This study provides key physical parameters for the application of TFHs in wearable medical devices and physiotherapy equipment.

## 1. Introduction

Electrothermal materials can convert electrical energy into thermal energy through the Joule heating effect (or resistive heating effect) when an electric current is applied [1,2,3]. In recent years, various electrothermal materials including metallic and non-metallic have been developed, yet they exhibit certain limitations in electrothermal performance and ductility [4,5]. Therefore, developing flexible electrothermal materials with both excellent electrothermal and ductility has become a research hotspot in the electric heating field. Initially, indium tin oxide (ITO) was widely used in fabricating electrothermal materials due to its high optical transmittance and good electrical conductivity [6,7], but its high brittleness, high cost, and poor wear resistance have restricted its widespread application [8,9,10]. Accordingly, alternative materials such as graphene (Gr) [11,12], carbon nanotubes (CNTs) [13,14,15], and silver nanowires (AgNWs) [16,17] have been investigated for use as electrothermal materials due to their favorable thermal and electrical conductivities [18]. Among them, CNTs have been extensively studied as promising replacements for traditional electrothermal materials because of their extraordinary properties [19,20,21]. CNTs are novel nanomaterials consisting of seamless, hollow cylindrical structures formed by rolling up graphene sheets of sp^2^-hybridized carbon atoms [22,23]. In particular, single-walled CNTs (SWCNTs) possess outstanding electrical, mechanical, and flexible properties, especially ultrahigh electron and hole mobilities [24,25]. At present, numerous studies have been conducted on transparent conductive films (TCFs) fabricated from SWCNT dispersions [26]. Nevertheless, owing to their large aspect ratio, CNTs tend to agglomerate in polymer matrices and deteriorate the dispersion uniformity, which necessitates complicated purification, separation and dispersion procedures [18]. Compared with multi-walled carbon nanotubes (MWCNTs), SWCNTs exhibit superior electrical conductivity, thinner thickness and more uniform morphology, yet possess relatively higher fabrication costs [27]. In electrothermal conversion applications, TCFs can produce far-infrared radiation (FIR) under electrical excitation [28] to achieve physiotherapeutic efficacy, and have therefore been used to prepare CNT flexible thin-film heaters (TFHs) with physiotherapeutic functions [29,30].

FIR refers to the invisible light beyond the red end of the visible spectrum and belongs to the electromagnetic wave category. It possesses skin-penetrating ability and strong thermal effects [31,32,33], and exhibits high therapeutic value for diseases characterized by inflammatory responses such as rheumatoid arthritis [31,34]. At present, specialized lamps and sauna devices capable of emitting pure FIR are commercially available, and FIR has become a safe, effective and widely used physiotherapy heat source [33]. Nevertheless, the heating technology of CNT flexible thin-film heaters (TFHs) has not been widely applied in the far-infrared physiotherapy industry, and relevant studies on the integration of CNT heating technology with physiotherapy are still insufficient. Currently, most commercial heating physiotherapy products adopt traditional technologies such as coil and moxibustion heating, which generally present drawbacks including poor safety, high risk of scalding and unsatisfactory physiotherapy efficacy [35]. Currently, SWCNT TFHs, acting as flexible heating elements, featuring outstanding flexibility, cuttability, acid resistance and mechanical fatigue resistance [36], can be used in heating wristband, heating knee pads, and heating clothing [37]. They exhibit abundant potential application scenarios in wearable medical devices and physiotherapy equipment [38,39]. Nevertheless, such flexible heaters still suffer from several drawbacks, including high power consumption and the contradictory trade-off between high optical transmittance and low sheet resistance [40,41].

In this study, two types of TFHs were fabricated via the bar coating method [42,43]. This coating process exhibits excellent compatibility with industrial mass production, enabling precise regulation of film thickness and suitability for large-scale fabrication. Nevertheless, it imposes stringent requirements on the stability of SWCNT dispersions [44]. The two types of TFHs differed in electrical resistance. By altering electrode connection configurations, the device resistance was effectively reduced. While high optical transmittance was maintained, the TFHs also presented regular morphology, which met the general application demands of physiotherapy instruments. Meanwhile, the devices displayed advantages including environmental friendliness, biosafety, excellent temperature uniformity and tunable electrothermal performance [29,45]. Furthermore, their fundamental physical properties and physiotherapy effects were tested and analyzed. Various characterization techniques were employed to evaluate the excellent comprehensive properties of the TFHs. The temperature drop rate and thermal variation of the TFHs attached to the arm were tested and compared with those of single TFHs. The results further verify that the FIR heat generated rapidly by the CNT TFHs can be absorbed by human skin and diffused throughout the body via blood circulation, providing key parameter support for its application in wearable medical devices and physiotherapy equipment.

## 2. Experimental

### 2.1. Materials

High-purity SWCNTs with a purity of 95 wt%, diameter of 2 nm, and length ranging from 5 to 30 μm were purchased from Tanxing High-Tech Co., Ltd. (Tianjin, China). PET films were supplied by Tianjin Wanhua Co., Ltd. (Tianjin, China). Sodium dodecyl benzene sulfonate (SDBS) was obtained from Shanghai Aladdin Biochemical Technology Co., Ltd. (Shanghai, China). All reagents and materials were used as received without further purification.

### 2.2. Fabrication Method and Process of TFHs

#### 2.2.1. Preparation of SWCNT Solution

Firstly, SWCNTs and SDBS were mixed in deionized water at a mass ratio of 1:10 to prepare CNT suspension with a concentration of 0.1 wt%. The mixture was subjected to water-bath sonication at 100 W for 2 h, followed by probe-type sonication at 120 W for 0.5 h. Subsequently, the SWCNT solution was centrifuged at 5000 rpm for 20 min. This process, including sonication and centrifugation, was repeated three times to remove incompletely dispersed CNT agglomerates from the suspension. Finally, 80% of the supernatant was extracted to obtain a relatively uniform CNT dispersion.

#### 2.2.2. Fabrication of SWCNT TCFs

Polyethylene terephthalate (PET) film substrates were cut into rectangles of 6 cm × 5 cm and 14 cm × 8.5 cm, immersed in deionized water, and sonicated to remove water-soluble impurities. The above procedure was repeated using ethanol in place of deionized water to further eliminate organic impurities. Then, the dried PET films were fixed on a bar coating plate at 80 °C. Approximately 10 mL of the SWCNT dispersion was dropped on the edge of the prepared PET substrate using a dropper, and a Mayer rod was applied at a coating speed of 25 mm/s to form a uniform liquid film with a thickness of 200 nm. Finally, the substrate was placed on a heating stage and thermally annealed at 120 °C for 10 min to prevent SWCNTs from detaching from the PET. After being removed from the heating stage, the TCFs were soaked, rinsed, and dried in deionized water to enhance the adhesion to PET substrate and remove impurities between the CNTs, and the water-washed TCFs were finally obtained.

#### 2.2.3. Fabrication of SWCNT TFHs

**Figure 1:**
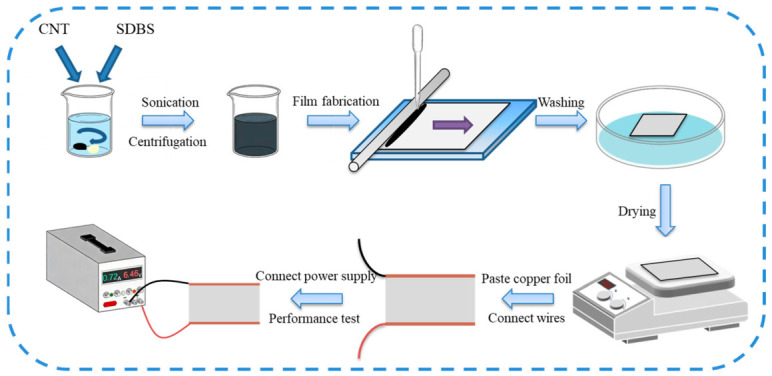
Preparation flow chart of TFHs.

On the edges of both sides of the transparent conductive films prepared above, a silver paste solution of about 3 mm was coated. After drying at 120 °C, copper conductive adhesive was pasted on the silver paste, and a layer of thermoplastic adhesive was coated on it. Then, it was heated and pressed to cover the PET film as a protective layer, forming a SWCNT TFH with complete structural functions. Welding wires to connect the voltage can realize heating. Figure 1 shows the preparation flow chart of TFHs.

### 2.3. Characterization

The surface morphology of TFHs was analyzed using a field-emission scanning electron microscope (FE-SEM, S4800, Hitachi, Tokyo, Japan). A four-probe tester (Keithley 2700 multimeter data acquisition system, Tektronics, Beaverton, OR, USA) was employed to measure the sheet resistance of TFHs. To evaluate the electrothermal behavior of TFHs, a direct current (DC) power supply (MS-605D, Maisen, Dongguan, China) was applied to both ends of the TFHs, and the current under different voltages was recorded. A thermocouple (DM6210-T07-R8T0S-0A-08 (M212), Maisen, Dongguan, China), which was fixed on the film surface with tape, was directly connected to a computer through an isolation converter to test the maximum surface temperature change of the electrothermal laminated film in real time. Thermal infrared (IR) images were recorded by an infrared camera (UTi220A, UNI-T, Maisen, Dongguan, China).

## 3. Results and Discussion

### 3.1. Characterization of SWCNT TFHs

**Figure 2:**
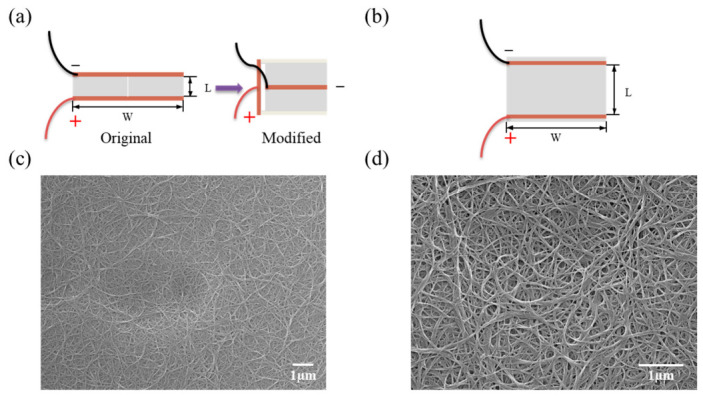
Schematic diagrams of TFH connections (**a**,**b**) and SEM images of TCFs at different magnifications (**c**,**d**): (**a**) TFH1; (**b**) TFH2; (**c**) 10,000× magnification; (**d**) 20,000× magnification.

For TFHs, the sheet resistance is the most dominant factor affecting their performance [46]. Therefore, the sheet resistances of two TFHs were measured and found to be similar. To prepare TFHs with different resistance values, two different electrode connection configurations were adopted, as illustrated in Figure 2a,b. TFH1 was a TFH with an area of 6 cm × 5 cm, while TFH2 had an area of 14.5 cm × 8 cm. Initially, reducing the L/W ratio was adopted to prepare TFHs with lower resistance, yet excessively slender device morphology obtained in this way cannot meet the application requirements of conventional medical devices. Accordingly, the electrode connection structure of TFH1 was employed to regulate the L/W ratio close to 1, which greatly expands its applicability in physiotherapy products. TFH1 adopted a parallel connection structure of two upper and lower heating films, which halved the width and doubled the length of the device. By comparison, TFH2 was fabricated in a conventional electrode connection mode. In addition, the as-prepared films support arbitrary cutting, enabling customized production according to diversified demands of medical devices.

The TFHs were characterized by scanning electron microscopy (SEM) to observe their micromorphology and structural features, followed by systematic analysis. The SEM images are shown in Figure 2c,d. It was observed that the CNTs exhibit a uniform and continuous interwoven network structure, tightly connected with each other via van der Waals forces, thus forming a highly interconnected three-dimensional conductive network. This uniformly distributed network effectively improved the overall electrical conductivity of the film. Meanwhile, numerous micro- to nanoscale pores were clearly observed and uniformly distributed within the network structure. These pores enabled multiple scattering and transmission in the TFHs, significantly enhancing the optical transmittance while preserving the integrity of the conductive network. The film exhibited a sheet resistance (*R_s_*) of 201.32 Ω/sq at an optical transmittance of 77.81% at 550 nm [46], and its FOM value calculated according to Equation (1) was 6.99. The synergistic effect of the porous and network structures endowed the TFHs with excellent light transmittance and electrical conductivity, endowing the films with transparent and unobstructed appearance to adapt to special application scenarios.

(1) $$FOM=\frac{\sigma_{DC}}{\sigma_{OP}}=\frac{188\left(\Omega \right)}{R_{s}}\frac{\sqrt{T}}{1-\sqrt{T}}$$

where *FOM* is the optoelectronic figure of merit, *σ_DC_* represents the direct current conductivity, *σ_OP_* denotes the optical conductivity, *R_s_* refers to the sheet resistance, and *T* is the visible light transmittance of the thin film.

### 3.2. Electrical Performance of SWCNT TFHs

**Figure 3:**
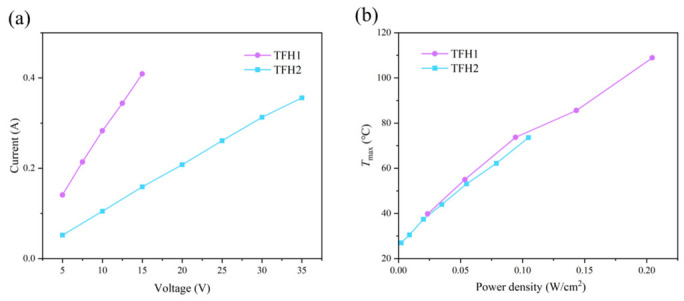
(**a**) Voltage–current curves of TFHs. (**b**) Relationship between power density and maximum temperature at various voltages for TFHs.

The current data were obtained by applying various voltages to the two TFHs within safe operating ranges (TFH1: 5–15 V, TFH2: 5–35 V), and the voltage–current curves were plotted as presented in Figure 3a. It was seen that the measured surface current of the two TFHs with different dimensions showed a linear variation with the applied voltage. Linear fitting was carried out accordingly, and the reciprocal of the fitted slope represented the resistance of the TFHs. The resistance of TFH1 was 37.49 Ω, and that of TFH2 was 97.83 Ω, indicating that for TFHs fabricated from the same material, a longer length and narrower width result in a lower resistance. The sheet resistance was further calculated using Equation (2). The sheet resistance of TFH1 was 208.48 Ω/sq, and that of TFH2 was 202.65 Ω/sq, which were relatively close to each other. This indicated that the TFHs achieved excellent synergistic matching of optoelectronic properties with high optical transmittance and low sheet resistance.

(2) $$R_{s}=R\frac{W}{L}$$

where *R_s_* is the sheet resistance of the TFHs, *R* is the resistance of the TFHs, *L* is the current transmission length of the TFHs, and *W* is the electrode length of the TFHs.

### 3.3. Response Time and Electrothermal Performances of SWCNT TFHs

**Figure 4:**
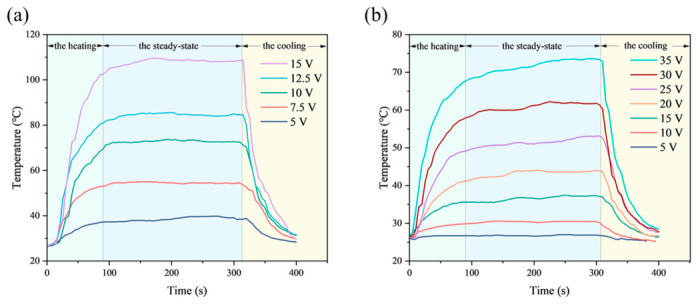
Time–temperature curves of TFHs under various voltages: (**a**) TFH1; (**b**) TFH2.

The temperature response time (defined as the time required for the temperature to rise from room temperature to the steady-state maximum temperature (*T*_max_)) was independent of the type and size of the heater, but depended on its resistance, the applied voltage, and the current passing through it [3]. Figure 4a,b shows the temperature evolution of TFH1 and TFH2 as a function of time under different applied voltages, respectively. At a given applied voltage, TFH1 exhibited a relatively high *T*_max_ due to its lower resistance. In addition, both TFHs could be rapidly heated and cooled, and the temperature basically reached *T*_max_ and remained to stable within 90–120 s, whereas the temperature of TFH2 rose slightly after reaching a stable state. When the power supply was turned off at 300 s, the temperatures of both TFHs dropped rapidly back to room temperature, and the time consumed for heating and cooling was roughly the same. For the same TFHs, *T*_max_ increased synchronously with the increase in applied voltage, indicating that their electrothermal performance can be effectively regulated by voltage.

Furthermore, to investigate the temperature response of the electrothermal films, the time-dependent temperature response curves were divided into three regions: the heating (0–100 s), the steady-state (100–300 s), and the cooling (after 300 s). Since TFHs were susceptible to external environmental influences, the heating time may increase or decrease accordingly. To achieve the same temperature, 10 V was applied to TFH1, while 35 V was required for TFH2, which indicated that TFH1 consumed less electric energy at the same temperature. Theoretically, the conversion from electrical energy to heat follows Joule’s law, *P* = *U*^2^/*R*, where *P* is the electric power, *U* is the input voltage, and *R* is the resistance. Therefore, as the L/W ratio of the TFHs increased, the resistance of the electrothermal film increased accordingly. To maintain the converted electrical energy and *T*_max_ approximately constant, the voltage was increased accordingly.

In the temperature steady-state region, according to the law of energy conservation, *T*_max_ remained constant under different applied voltages, that is, the thermal gain from the input power was equal to the heat loss via convection and radiation. The electrothermal conversion efficiency was expressed by the heat transfer coefficient *h*_r+c_ contributed by radiation and convection:

(3) $$h_{r+c}=\frac{T_{m}-T_{0}}{I_{c}U_{0}}$$

where *I*_C_ is the steady-state current, *U*_0_ is the applied voltage, *T*_m_ is the maximum temperature, and *T*_0_ is the initial temperature. If an electrothermal material achieves a high temperature rise (*T*_m_ − *T*_0_) at low power consumption (*I*_C_*U*_0_), it will exhibit a high *h*_r+c_ value and demonstrate high electrothermal conversion efficiency [18,47].

According to Equation (3), the value of *h*_r+c_ for TFH1 was 16.71 °C/W, while that for TFH2 was 4.29 °C/W. The higher value of TFH1 was attributed to its lower resistance, which enabled higher Joule heating output power under the same voltage [18,48]. In addition, the temperature of the electrothermal film increased steadily with increasing applied voltage, enabling its application in heating and physiotherapy products to realize rapid and stable temperature control.

Figure 3b illustrates the relationship between the power density and the maximum temperature *T*_max_ at various applied voltages for the two TFHs. The desired *T*_max_ can be effectively controlled by adjusting the applied power density. When the two TFHs reached an identical power density, their steady-state maximum temperatures were close to each other. However, to achieve the same maximum temperature, TFH2 required a higher applied voltage to raise its power density accordingly. In addition, the variation trends of *T*_max_ with power density were highly consistent between the two samples, indicating that they possessed similar thermal response characteristics and there was no essential difference in electrothermal conversion efficiency. Combined with the structural characteristics of TFHs, higher power density remarkably improved the steady-state heating temperature of devices. Meanwhile, TFHs fabricated by the same process with a smaller L/W ratio exhibited lower resistance, higher input power density under identical voltage, and more prominent electrothermal heating performance.

**Figure 5:**
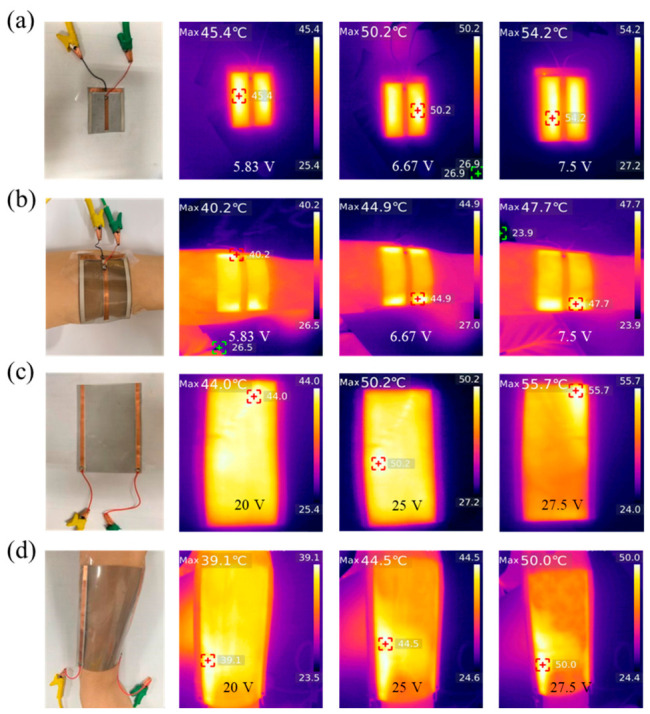
Infrared images of TFHs. (**a**) Optical image of TFH1 with electrodes and corresponding infrared images at 5.83 V, 6.67 V, and 7.5 V, respectively; (**b**) optical image of TFH1 attached to an arm and corresponding infrared images on the arm at 5.83 V, 6.67 V, and 7.5 V, respectively; (**c**) optical image of TFH2 with electrodes and corresponding infrared images at 20 V, 25 V, and 27.5 V; (**d**) optical image of TFH2 attached to an arm and corresponding infrared images on the arm at 20 V, 25 V, and 27.5 V.

To further test the electrothermal performance of TFHs, experiments were conducted within the temperature range acceptable to human skin (45~55 °C). The voltage applied to the TFHs was adjusted according to the set temperatures (45, 50, and 55 °C), and after the temperature readings stabilized, images were captured using an infrared camera, as shown in Figure 5a,c. The images show relatively uniform color distribution on the TFHs, attributed to the good distribution of SWCNTs [45]. The TFHs were then attached to the arm, as shown in Figure 5b,d, causing the TFHs’ temperature to decrease. After the readings stabilized again, the temperature and its distribution were recorded with the infrared camera. During testing, the TFHs maintained close contact with the skin, also demonstrating their bending resistance [10,49]. In addition, bending cycle tests were carried out for further analysis. The results showed that the sheet resistance of TCFs increased by no more than 7.1% after 5000 bending cycles, which verified the excellent flexibility and mechanical properties of the films [20].

After multiple tests of the two TFHs at different initial temperatures, the temperature of the films decreased by approximately 5 °C after attached to the arm and then remained stable, proving that the heat generated by the TFHs under applied voltage can be absorbed by the human body and dissipated with blood circulation. The color distribution on the films remained basically uniform. As the applied voltage increased, the color of the infrared images of the CNTs changed accordingly, and the background color around the TFHs became darker, indicating that the film temperature gradually increased. This confirmed the excellent electrical and thermal conductivity of the CNTs, as well as the high uniformity of the CNT layer. The above results demonstrate that the TFHs possessed outstanding electrothermal performance, and the electrothermal capability of CNTs can be effectively controlled by adjusting the applied voltage.

### 3.4. Heat Transfer Analysis of SWCNT TFHs

**Figure 6:**
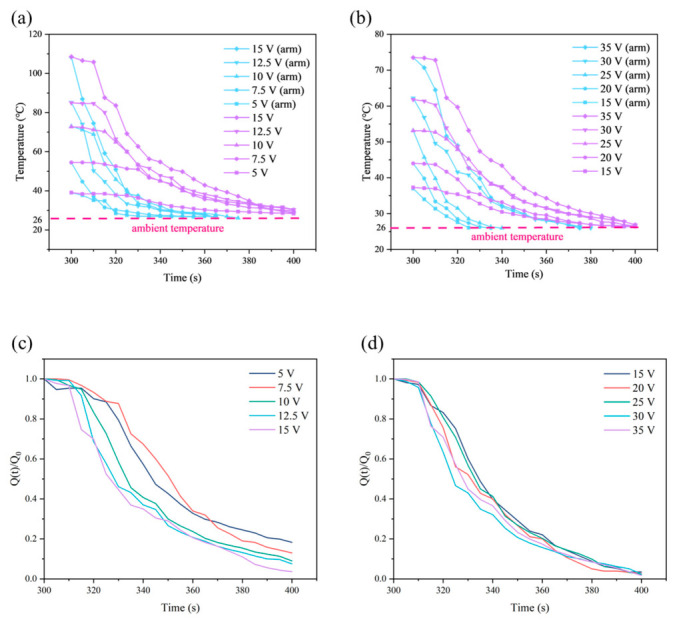
Comparison of temperature drop rates of TFHs with and without attachment to arm skin: (**a**) TFH1; (**b**) TFH2. Dimensionless residual heat–time curves of TFHs: (**c**) TFH1; (**d**) TFH2.

After measuring the surface temperature of the TFHs, the temperature drop rate of the TFHs after voltage removal was further analyzed. The TFHs were positioned approximately 1 cm away from the arm to avoid skin burns at high temperatures rather than being in full contact. Figure 6a,b shows the comparison between the temperature drop rate of TFHs in air and that when attached to the skin. It can be seen that the TFHs placed near the arm exhibited a faster temperature drop rate, indicating that the TFHs can rapidly transfer heat to the human body. This further verified that the FIR waves emitted by the TFHs in the form of heat release can be effectively absorbed by human skin. According to Equation (4), the temperature drop rate of the TFHs was evaluated by calculating the ratio of heat released after voltage removal, and the dimensionless residual heat–time curves of TFHs were plotted in Figure 6c,d. The figures revealed that a higher applied voltage led to a higher maximum temperature, which resulted in a faster temperature drop rate after power cut off. All curves under different voltages showed an exponential decay trend, which is consistent with Newton’s law of cooling and also agrees with the decreasing trend of the temperature drop rate in Figure 6a,b.

(4) $$\frac{\;Q(t)}{Q_{0}}=\frac{T\left(t\right)-T_{e}}{T_{0}-T_{e}}$$

where $Q\left(t\right)=Cm\left(T\left(t\right)-T_{e}\right)$ represents the heat at time *t* (relative to the environment), $Q_{0}=Cm\left(T_{0}-T_{e}\right)$ is the initial heat, *T*(*t*) is the temperature of the TFH at time *t*, *T_e_* is the ambient temperature (constant), which is 26 °C, and *T*_0_ is the initial temperature.

### 3.5. Heat Transfer Mechanism of SWCNT TFHs on Human Skin

**Figure 7:**
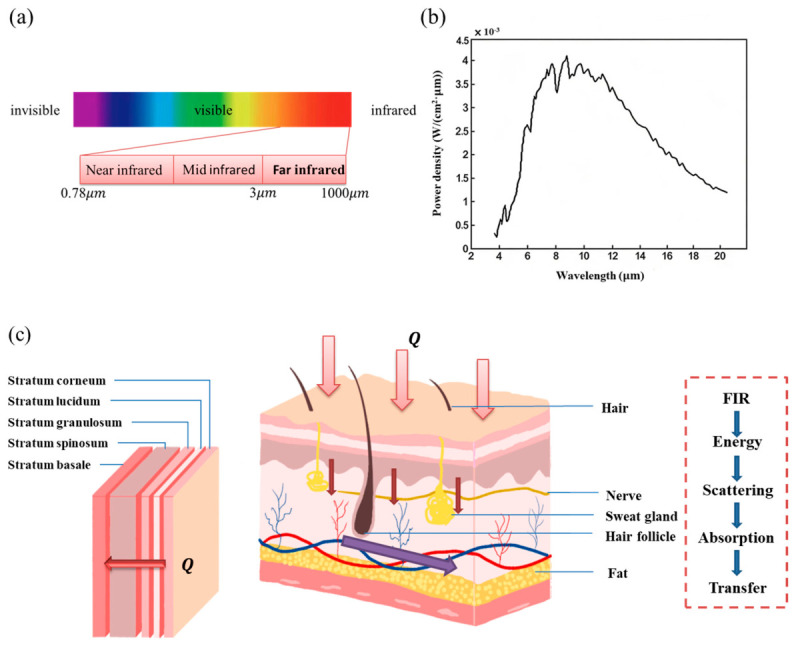
Schematic illustration of heat transfer mechanism of TFHs acting on human skin: (**a**) light spectra, (**b**) FIR emission test of TFHs, (**c**) mechanism of FIR interacting with skin.

Figure 7a shows the schematic diagram of the heat transfer mechanism of TFHs acting on the human skin. Figure 7a shows the light spectrum. FIR is a specific band in the infrared electromagnetic spectrum. According to wavelength classification, the infrared spectrum consists of three regions: near-infrared, mid-infrared, and far-infrared. Most studies reported that the wavelength range of FIR was between 3 and 1000 µm [31,33,50]. In the infrared spectrum, FIR transferred energy in the form of thermal energy, which was perceived by skin thermoreceptors, penetrated subcutaneous tissues, and acted on deep tissues to generate heat. Figure 7b presents the FIR spectrum of TFHs tested by the National Institute of Measurement and Testing Technology. It can be observed that the wavelength intensity peak of the far-infrared waves emitted by the electrified TFHs is centered at 8–12 µm, which falls within the peak range of 6–14 µm which can be easily absorbed by the human body [31,51,52], confirming that the waves emitted by TFHs are absorbable by the human body. Figure 7c illustrates the mechanism of FIR interacting with the skin, indicating that FIR energy can penetrate subcutaneous tissues to a certain depth, with a penetration potential of approximately 1.5 inches below the skin [53]. FIR acted on deep tissues to generate and dissipate heat, triggering organic responses. The heat was finally absorbed by the human body through blood circulation, achieving a certain physiotherapeutic efficacy.

## 4. Conclusions

This study focused on the application potential of SWCNT TFHs in medical physiotherapy. Two TFHs with different electrode connection modes were prepared via the bar coating method, and their fundamental physical properties and physiotherapeutic performance were systematically tested and analyzed. It was found that the synergistic effect of the porous and network structures of TFHs endowed them with excellent optical transmittance and electrical conductivity. TFHs exhibited uniform and rapid FIR and rapid temperature rise and fall capabilities at low voltages. At the same voltage of 10 V, the temperature rise per unit input power for TFH1 was 16.71 °C/W, while that of TFH2 was 4.29 °C/W. Meanwhile, the variation laws of *T*_max_ versus power density for the two samples were highly similar. This demonstrated that TFH1 and TFH2 prepared by the same TCFs both possessed excellent and efficient electrothermal conversion efficiency as well as outstanding comprehensive electrothermal properties. In addition, it verified that a smaller L/W ratio corresponded to lower resistance, resulting in stronger Joule thermal effect under the same applied voltage. After the temperature became stable, the temperature of the TFHs dropped by approximately 5 °C when attached to the human arm, and the film cooled down more rapidly after the voltage was removed, proving that the heat generated by the energized TFHs could be absorbed by the human body. Meanwhile, the peak wavelength of FIR emitted by the TFHs was 8–12 µm, which fell within the infrared band (6–14 µm) that was easily absorbable by the human body. This further verified that the energy generated by TFHs can be absorbed by human skin and diffused throughout the body via blood circulation, enabling application in heating physiotherapy products to achieve a certain physiotherapeutic effect. In summary, this study provides key physical parameter support for the application of flexible CNT TFHs in wearable medical and physiotherapy devices, and confirms their advantages as an ideal material for the heating physiotherapy industry.

## Data Availability

The original contributions presented in this study are included in the article. Further inquiries can be directed to the corresponding authors.
